# The development of an analytical method to evaluate the nitrosamine profile in cooked ham with different preservatives and in rat feces fed with them

**DOI:** 10.1007/s00216-025-06214-2

**Published:** 2025-11-23

**Authors:** Claudia Giménez-Campillo, Yolanda Guerrero-Núñez, Natalia Campillo, Natalia Arroyo-Manzanares, Isidro Guillén, Pascuali Vizcaíno, Carlos de Torre-Minguela, Pilar Viñas

**Affiliations:** 1 Department of Analytical Chemistry, Faculty of Chemistry, Regional Campus of International Excellence “Campus Mare Nostrum”, University of Murcia, 30100 Murcia, Spain; 2 Cátedra PROSUR de Biotecnología de Alimentos, University of Murcia, 30100 Murcia, Spain; 3 Department of Research and Development, PROSUR S.L., Av. Francisco Salzillo, P/27-2, San Ginés, 30169 Murcia, Spain

**Keywords:** Nitrosamines, Polyphenols, Nitrite, Animal model, Feces, Liquid chromatography-mass spectrometry

## Abstract

Nitrosamines are classified as carcinogenic chemicals and are a growing public health concern. They have been widely documented in food, which is the main route of human exposure. However, there is little research on their appearance in biological samples, such as feces. This study presents a methodology for determining the concentration of 14 nitrosamines in cooked hams and fecal samples obtained from animals fed with them, allowing changes in the nitrosamine concentration during digestion to be identified. The novelty of this research lies in the fact that it is the first to evaluate whether incorporating polyphenols into cooked ham influences the levels of NAs in feces using an animal model. The proposed analytical method based on ultrasound-assisted extraction of the nitrosamine high-performance liquid chromatography and mass spectrometry using a triple quadrupole achieved low limits of quantification (in the 0.44–47 ng g^−1^ range), good precision (coefficient of variation of less than 12.4%), and trueness for both matrices. A total of 68 samples were analyzed, with five nitrosamines detected in the cooked ham samples and seven in the fecal samples. Statistical analysis revealed that the nitrosamine profile mainly depended on the biological matrix. While the preservative did not significantly affect the nitrosamine profile in cooked ham, it did modulate the nitrosamine concentration in feces. Specifically, diets containing polyphenols significantly reduced fecal nitrosamine levels compared to diets containing nitrites, suggesting a possible protective role against the formation of these compounds in the gut.

**Graphical Abstract:**

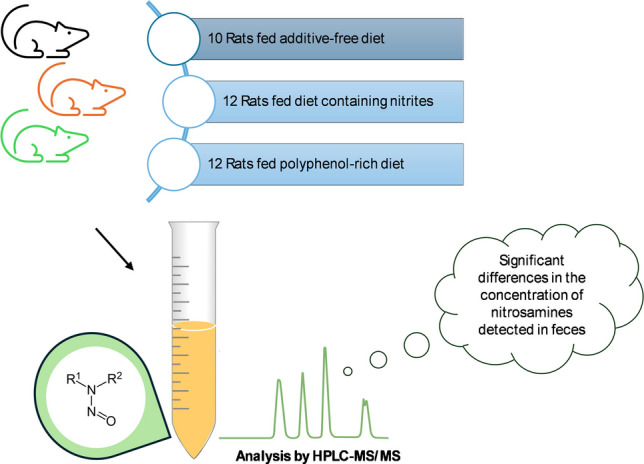

**Supplementary Information:**

The online version contains supplementary material available at 10.1007/s00216-025-06214-2.

## Introduction

Nitrosamines (NAs) are organic compounds formed by the reaction of a nitrosating group, such as nitrates, nitrites, or nitrous oxide, with an amine or amide [1]. To date, more than 300 volatile and non-volatile NAs have been described, all of which share a common structure (N–N = O) [2]. Twenty-four of these NAs have been classified by the International Agency for Research on Cancer (IARC) according to their potential to cause cancer in humans, with N-nitrosodimethylamine (NDMA) and N-nitrosodiethylamine (NDEA) being classified as potentially carcinogenic to humans (Group 2 A) and N-nitrosoethylmethylamine (NEMA), N-nitrosodi-n-propylamine (NDPA), N-nitrosodi-n-butylamine (NDBA), N-nitrosopiperidine (NPIP), N-nitrosomorpholine (NMOR), N-nitrosopyrrolidine (NPYR), N-nitrosodiethanolamine (NDELA), and N-nitrososarcosine (NSAR) as compounds possibly carcinogenic to humans (Group 2B) [3].

The European Food Safety Authority (EFSA) recently published a report warning of the risks to human health associated with the consumption of foods containing NAs [4]. Although this report only assessed the risk of NAs present in food, it stated that various N-nitroso compounds had been detected in biological fluids such as blood, urine, breast milk, and gastric juices, without specifying their possible origin, but did not rule out the possibility of endogenous formation [5]. This endogenous process can occur during digestion, as the acidic pH of the stomach favors the nitrosation of amines present in ingested food [6]. This statement caused great alarm among the population, since in recent years the presence of these chemical contaminants has been detected in various matrices such as food [7], beverages [8], tobacco [9], personal care products [10], pharmaceuticals [11], and environmental samples [12]. In addition, after learning of their high toxicity, several studies have shown that exposure to NAs increases the risk of certain types of cancer, such as cancer of the esophagus, stomach, colon, liver, and lung [13, 14].

In recent years, most studies have focused on detecting and quantifying NAs in food because diet is considered the primary source of human exposure to these compounds. The highest levels have been found in processed meat, cheese, smoked fish, beer, and canned vegetables [15]. The high presence of these compounds in processed meat products has been associated with the use of nitrites as food additives. These additives react with amines present in the meat to favor the formation of these compounds, especially during intense thermal processes, such as cooking or reheating [16, 17]. Based on this information, organizations such as IARC and the World Health Organization (WHO) have classified certain processed meat products as carcinogenic and established maximum permissible limits for NAs in food [18]. In response to these concerns, recent research has explored replacing these additives with natural alternatives, such as additives rich in polyphenols, flavonoids, or other active compounds that have certain health benefits in addition to their antimicrobial effects [19, 20].

Despite growing evidence for the presence of NAs in meat-based foods [21–24], there are very few studies on the presence of these compounds in biological samples. Few studies have analyzed feces after the ingestion of nitrite-rich foods, despite the gastrointestinal tract being one of the main sites where endogenous formation could occur [15]. Previous studies have suggested that fecal samples and human microbiota do not contain NAs [25, 26]. However, a more recent study suggests limitations in the sample preparation that affect the analytical outcome. The application of an inappropriate extraction step and the presence of numerous interferents (fatty acids, amines, inorganic salts, etc.) in the samples compromised the sensitivity and specificity of the method, which could have led to erroneous results [27].

In this context, the use of animal models has become very relevant, as it allows for simulating in a controlled way the ingestion of precursor compounds and to accurately study the formation, absorption, metabolism, and excretion of NAs in different biological matrices [28–30]. These models are useful for confirming the endogenous formation of NAs, establishing possible dose–response relationships, and validating robust analytical methodologies. Additionally, they overcome some of the ethical and technical limitations associated with human sampling [31, 32]. Therefore, it is crucial to analyze food and biological matrices together because a complete risk assessment requires knowledge of not only the NAs in ingested food, but also their possible endogenous formation. This integrated approach is key to improving the understanding of the real impact of these compounds on human health.

The most commonly used technique for the determination of volatile NAs is gas chromatography coupled to mass spectrometry (GC-MS) [1, 8, 10, 11, 17, 21–24, 27, 33–35], although it has proven several limitations for the determination of certain less volatile NAs and for the identification of many polar NAs. On the other hand, high-performance liquid chromatography coupled to tandem mass spectrometry (HPLC-MS/MS) offers advantages [7, 36–38] and opens the possibility to determine a wider range of NAs [36, 39].

The objective of this study is to develop and evaluate a new analytical methodology for determining 14 NAs in two different biological matrices (cooked ham and fecal samples). To achieve this purpose, ultrasound-assisted extraction (UAE) was employed to improve sample preparation and HPLC-APCI-QqQ-MS/MS for its quantification. This methodology was applied to determine the NA content in three types of cooked ham (prepared without preservatives, with nitrites, or with polyphenols (a nitrite substitute)) and in fecal samples obtained from animals fed these three types of cooked ham. The challenge was to assess whether the type of preservative influences the NA content in food and after digestion, establishing possible relationships between the different preservatives used and the levels of NAs detected in both cooked ham and fecal samples, without evaluating subsequent biological effects. This is the first time that research has been conducted to determine whether the addition of polyphenols to processed meats can influence NA levels in fecal samples using an in vivo animal model.

## Materials and methods

### Reagents

An EPA 8270/Appendix IX certified reference material containing nine NAs: NDMA, NEMA, NDEA, NDPA, NDBA, NMOR, NPIP, NPYR, and nitrosodiphenylamine (NDPhA) in methanol at a concentration of 2000 µg mL^−1^ was supplied by Sigma Aldrich (St. Louis, MO, USA). The remaining standards (N-nitrosomethylphenylamine (NMPhA), nitrosoethylphenylamine (NEPhA), nitrosodiisobutylamine (NDiBA), N-nitrosodicyclohexylamine (NDCHA), and nitrosodibenzylamine (NDBzA)) were provided individually by Toronto Research Chemicals (North York, Ontario, Canada). Individual solutions were prepared in methanol at a concentration of 1000 µg mL^−1^. All concentrated solutions were stored in a freezer (−20 °C) and in the absence of light. In addition, a standard solution containing the 14 NAs was prepared daily in ultrapure water at a concentration of 1 µg mL^−1^.

The polyphenol-rich extract was supplied by PROSUR SL (Murcia, Spain). LC-MS grade ultrapure water and MS grade formic acid were provided by Scharlau (Barcelona, Spain). LC-MS grade methanol was supplied by VWR International (Radnor, Pennsylvania, USA), while ammonium formate was obtained from Thermo Scientific (Madrid, Spain). Finally, chloroform was purchased from Sigma Aldrich and trichloroacetic acid (TCA) from Riedel-de-Häen (Wunstorf, Germany).

### Animal model

A total of 34 rats (F344/DuCrl), aged 4–5 weeks, were provided by Charles River Laboratories (Saint-Germain-sur-l’Arbresle, France). Rats were pair-housed in cages under controlled temperature and humidity conditions with a 12-h light/dark cycle, in accordance with animal experimentation guidelines because rats are social animals, and solitary confinement severely impacts their behavior and well-being. The animals were housed in the Animal Facility of the University of Murcia (authorized facility No. ES 300305440012). The experiments associated with this research were approved by the Ethical Committee for Animal Experimentation of the University of Murcia (authorization code: A13211206) in accordance with the provisions of EU Directive 2010/63/EU on the protection of animals used for scientific purposes. The animals were then randomly assigned to three groups (nitrite-free diet (control group) *n* = 10, nitrite diet *n* = 12, and polyphenol diet *n* = 12). Each study group was fed a specific diet (meat diet without nitrites, meat diet with nitrites, or meat diet with polyphenols). Between days 83 and 85, each animal was placed in a metabolic cage and feces were collected. These biological samples were stored at a temperature of −20 °C. On day 93, in a random order, the rats were euthanized by CO_2_ asphyxiation.

### Animal diets

The experimental diets were prepared on the basis of a modified AIN76 diet in the form of a mixture with cooked ham, as described by Santarelli et al. [40]. The base diets used in the cooked ham diets were manufactured by Research Diets Inc. (New Brunswick, NJ) and were balanced for iron, protein and fat. The composition of the ham-free basal diet was (g/100 g): sucrose, 27.8; casein, 5.0; maize starch, 5.0; safflower oil, 5.00; cellulose, 5.0; S10001 calcium-free mineral mix, 3.5; dicalcium phosphate, 0.21; DL-methionine, 0.3; V10001 vitamin mix, 1.0; choline bitartrate, 0.20.

The cooked hams have been prepared from the meat of the pig shoulder in the PROSUR Meat Laboratory (Murcia, Spain). Different curing mixtures were used to elaborate the cooked hams. The cooked ham without nitrites was made with a brine consisting of 1.8 g of sodium chloride and 0.03 g of sodium ascorbate per 100 g of meat. The cooked ham with nitrites was cured with 0.012 g of sodium nitrite (equivalent to 120 mg kg^−1^ NaNO_2_) in addition to the above-mentioned components. Finally, the cooked ham with polyphenols was elaborated with a mixture of 1.4 g of sodium chloride and 1 g of an extract rich in polyphenols (NATPRE T-10 HT S) per 100 g of meat. The exact composition of the polyphenol mixture used is not disclosed, as this information is protected by the supplier’s trade secrets. The hams were then cooked up to an internal temperature of 68 °C in an oven with a maximum temperature of 73–75 °C. After cooking, the hams, each weighing approximately 1.3 kg, were vacuum-packed in low oxygen permeability plastic bags and frozen at −20 °C. Prior to inclusion in the diets, the cooked ham slices were thawed and exposed to air, in the dark, at a temperature of 4 °C, for a period of 5 days, according to the oxidation protocol proposed by Santarelli et al. [40].

To prepare the different diets, 53 g of the modified AIN76 diet was combined with 187 g of oxidated ham. For the meat diet without nitrites, oxidated ham without nitrites was used; for the meat diet with nitrites, oxidated ham with nitrites was used; and for the meat diet with polyphenols, oxidated ham with polyphenols was used.

### Samples

A total of 68 samples were collected: 20 of the group of rats fed with nitrite-free ham (10 ham samples and 10 fecal samples), 24 of the group of rats fed ham with nitrites (12 ham samples and 12 fecal samples), and 24 of the group fed ham with polyphenols (12 ham samples and 12 fecal samples).

### Instrumentation

An ExionLC system in combination with a QTRAP 6500 + mass spectrometer detector provided with an atmospheric pressure chemical ionization source (HPLC-APCI-QqQ-MS/MS) from Sciex (Framingham, USA) was used for the analysis of NAs. The APCI source was operated in positive mode. An ACQUITY UPLC HSS T3 chromatographic column with a pore size of 100 Å, a particle size of 1.8 µm, an internal diameter of 2.1 mm, and a length of 100 mm from Waters (Barcelona, Spain) was employed for the chromatographic separation at 40 °C. Multiple reaction monitoring (MRM) mode was selected for MS data, while the SCIEX OS software, version 3.3.0.12027, was used for the identification and quantification of NAs.

An UP 200H ultrasonic probe processor (Dr Hielscher, Teltow, Germany) equipped with a titanium sonotrode (7 mm internal diameter) and operating at 200 W in liquid medium was used to extract NAs from the matrix. In addition, a centrifuge of type MPW-150R (Warsaw, Poland) was used for the preparation of the samples. After centrifugation, the extracts obtained were filtered using 1-mL needleless Nipro syringes and 25-mm diameter 0.2-µm pore nylon filters supplied by Agilent Technologies (Santa Clara, CA, USA).

### Sample treatment and HPLC‑APCI-QqQ‑MS/MS analysis

The analytical procedure used to prepare the fecal and cooked ham samples consisted of weighing 0.3 g of feces or 0.1 g of ham into 15-mL falcon tubes. Then, 5 mL of a 5% v/v formic acid solution was added to each tube. The tubes were then placed in an ice bath and subjected to ultrasonic vibrations for 5 min. These vibrations, produced by an ultrasonic probe emitting 0.75 s pulses with an amplitude of 105 µm, helped to release the NAs present in the ham and fecal matrices. Subsequently, the mixtures were centrifuged for 5 min at 10 °C and 6000 rpm (3420 g). Finally, the resulting supernatants were filtered through 0.2-µm nylon filters and a volume of 50 µL was injected into the HPLC instrument [41].

A two-solvent mobile phase was used for the chromatographic separation. Solvent A was a 0.1% v/v formic acid aqueous solution containing 5 mM ammonium formate, while solvent B was methanol with 0.1% v/v formic acid and 5 mM ammonium formate. The mobile phase flow rate was set at 0.4 mL min^−1^. The elution program started at 5% B and was maintained for 0.5 min. The percentage of B was then increased to 15% after 0.5 min and maintained for 1 min. The percentage of B then increased linearly to 65% in 4 min and was maintained for 3 min. Next, the percentage of B increased to 95% in 1 min and was maintained for another minute. Finally, it decreased to 5% B in 0.5 min and remained at this percentage for 3.5 min, giving a total analysis time of 15 min. Values between 8 and 64 V were applied for the collision energies (CE), while the declusting potential (DP) values for all NAs were between 36 and 120 V. The ionization source temperature was 300 °C, while the capillary voltage was 5500 V. The determination of NAs was performed using the MRM transitions listed in Table 1.

**Table 1:** HPLC-QqQ-MS/MS conditions for the studied NAs

AnalyteAQ	Molecular formulaAQ	RT^a^(min)AQ	Precursor ions (m/z)AQ	Product ions^b^ (m/z)AQ
NDMAAQ	(CH3)2N2OAQ	1.62AQ	75.0AQ	43.1 (Q)AQ 58.0 (q)AQ
NMORAQ	C4H8N2O2AQ	2.50AQ	117.0AQ	87.2 (Q)AQ 86.2 (q)AQ
NEMAAQ	C3H8N2OAQ	2.95AQ	89.0AQ	60.9 (Q)AQ 43.0 (q)AQ
NPYRAQ	C4H8N2OAQ	3.06AQ	100.9AQ	55.1 (Q)AQ 59.0 (q)AQ
NDEAAQ	(C2H5)2N2OAQ	4.75AQ	103.0AQ	75.2 (Q)AQ 47.1 (q)AQ
NPIPAQ	C5H10N2OAQ	5.16AQ	115.0AQ	69.1 (Q)AQ 41.2 (q)AQ
NMPhAAQ	C7H8N2OAQ	6.82AQ	136.9AQ	66.0 (Q)AQ 107.1 (q)AQ
NDPAAQ	(C3H7)2N2OAQ	7.02AQ	131.2AQ	89.2 (Q)AQ 43.3 (q)AQ
NEPhAAQ	C8H10N2OAQ	7.50AQ	150.9AQ 121.0AQ	77.1 (Q)AQ 106.0 (q)AQ
NDBAAQ	(C4H9)2N2OAQ	8.66AQ	159.1AQ	103.1 (Q)AQ 57.0 (q)AQ
NDiBAAQ	(C4H9)2N2OAQ	8.89AQ	159.1AQ	57.0 (Q)AQ 103.1 (q)AQ
NDPhAAQ	(C6H5)2N2OAQ	9.26AQ	170.1AQ	93.0 (Q)AQ 92.0 (q)AQ
NDBzAAQ	(C7H7)2N2OAQ	10.12AQ	227.1AQ	91.0 (Q)AQ 65.0 (q)AQ
NDCHAAQ	(C6H11)2N2OAQ	11.23AQ	83.1AQ 129.1AQ	55.1 (Q)AQ 83.1 (q)AQ

^a^RT, retention time

^b^Q, quantitative ion; q, qualitative ion

### Method validation procedure

The proposed analytical method for determining fourteen NAs in cooked ham and fecal samples was validated in accordance with International Council for Harmonisation (ICH) guideline M10 on the validation of bioanalytical methods [42]. Matrix effect, linearity, limits of detection (LODs) and quantification (LOQs), precision, trueness, stability, and carry-over were evaluated.

Two calibration curves were generated to investigate whether there was a matrix effect: one in the absence of matrix (with the aqueous standards in the 0.5–50 ng mL^−1^ range) and the other in its presence, in a concentration range of 5–500 ng g^−1^. These studies were carried out using a sample of cooked ham made with polyphenols, as well as fecal samples from a rat that had been fed this ham. After constructing the fourteen calibration lines, their slopes were compared using an ANOVA statistical test. This test determines whether there are significant differences in the slopes for each analyte. If the *P*-value > 0.05, it is assumed that there are no significant differences, and quantification can be performed using the aqueous calibration. However, if the *P*-value < 0.05, it is concluded that there are significant differences, and therefore, quantification would be carried out in the presence of the matrix.

The next step was to determine whether there were significant differences within each of the analyzed matrices (ham or fecal samples). For this purpose, two concentration levels (25 and 200 ng g^−1^, except for NDMA which was performed at 200 and 500 ng g^−1^) of six different samples per matrix (2 for each group of ham or rats) were analyzed in triplicate. Next, the relative standard deviation (RSD) was determined for each NA at the two evaluated concentrations. According to the validation guide, if the RSD is less than 15%, there are deemed to be no significant differences between the analyzed matrices, and calibration can be performed using a model matrix. Conversely, if the RSD exceeds 15%, the differences are considered statistically significant, and each sample must be quantified using its own calibration curve.

Fourteen calibration curves, one for each analyte, were generated to assess the linearity of the method in both matrices. For this purpose, matrices of cooked ham and rat feces, which had previously been characterized as free of NA, were spiked at eight different concentrations. In order to broaden the scope of the study, particularly with regard to less sensitive NAs, the upper limit was increased to 1000 ng g^−1^. To construct calibration curves, the analytical signal obtained was plotted against the corresponding concentration.

The sensitivity of the method was investigated by determining LODs and LOQs values, defined as the concentrations giving signals three (S/N = 3) and ten (S/N = 10) times greater than the noise [43].

To determine the precision and trueness, six different samples of each matrix were spiked with NAs at four concentrations (5, 25, 200, and 400 ng g^−1^, except for NDMA which were spiked at 200, 400, 600, and 800 ng g^−1^) and analyzed using the proposed analytical procedure. These samples were analyzed five times on the same day (*n* = 30), and the procedure was repeated on three consecutive days (*n* = 90).

The stability of the standards and sample solutions was also investigated. Stability studies of the standard solutions of the 14 NAs in water (50 ng mL^−1^) were evaluated at 24 and 48 h in triplicate. On the other hand, the stability of the cooked ham and fecal sample solutions containing NAs at a concentration of 200 ng g^−1^ was evaluated in three samples per matrix (one for each study group). Each sample was analyzed in triplicate after being stored at 25 °C for 24 or 48 h.

Finally, carry-over studies were performed. Several blanks (non-fortified samples) were analyzed following the analysis of a fortified sample of feces at 500 ng g^−1^, and the variation in analytical signals (RSD) was studied.

### Statistical analysis

The data were analyzed statistically using the following software: PAST (version 4.3, Oslo, Norway) and SigmaPlot (version 15.0, San Jose, CA, USA).

First, a two-way PERMANOVA analysis was performed using PAST software to simultaneously compare NA concentration levels in different matrices (cooked ham and feces) and differentiate between three study groups: no preservatives, with nitrites, and with polyphenols. The analysis determined if there were significant differences in the NA profile between the matrices and preservative types and if there was an interaction between these two factors. A threshold value of *P*-value = 0.05 was set for PERMANOVA statistical tests. A *P*-value below 0.05 indicates significant differences between study groups, while values above 0.05 suggest no significant differences.

A one-way PERMANOVA was then applied to each matrix to assess overall differences in NA profiles between the three study groups. If significant differences were detected, a univariate analysis was performed using the Kruskal–Wallis test for each NA, as the data did not follow a normal distribution. This approach allowed for the identification of the specific compounds contributing to the overall differences detected by PERMANOVA. Additionally, a Dunn test was performed to identify among which specific groups the difference detected by the Kruskal–Wallis test was found. Both statistical tests were performed using SigmaPlot software. Note that the univariate analysis was only applied to the matrix in which the one-way PERMANOVA analysis revealed significant differences.

Finally, to determine if the presence of an NA in one matrix was related to its presence in the other, 2 × 2 contingency tables were constructed. These tables collected cases in which each compound was present in both matrices, in one matrix only, or in neither. Fisher’s exact test was then applied to these tables to analyze whether there was a statistically significant relationship between the matrices, i.e., whether the detection of an NA in one matrix was associated with the detection of the same NA in the other. When an NA was detected in only one matrix, generating zero frequencies in the table and limiting the applicability of Fisher’s test, the *Z*-test was used as an alternative. This analysis assessed whether the uneven distribution of the compound between the matrices was statistically significant. As with the PERMANOVA tests, the *P*-value threshold was set at 0.05. Thus, when the *Z*-test result was lower than 0.05, the test concluded that there were significant differences for both matrices. Conversely, if the *P*-value was above 0.05, there were no significant differences.

## Results and discussion

### Study of HPLC‑APCI-QqQ‑MS/MS conditions

Optimization of the method started with the individual infusion of the available NAs standards at a concentration of 1 µg mL^−1^ into the mass spectrometer, being ionized in positive mode. During this process, the MRM transitions were optimized for each analyte, as well as the parameters DP and CE. For those analytes for which individual standards were not available, technical specifications provided by the instrument manufacturer and literature references were consulted to obtain the appropriate MRM transitions [37, 41, 44]. The DP and EC values were then evaluated by additional tests to determine the optimal values offering the best resolution and sensitivity for the analytes. Finally, DP values between 36 and 120 V and EC values between 8 and 64 V, depending on the NA, were selected.

The next step was to select the conditions to achieve correct separation of the 14 NAs studied. The chromatographic column, mobile phase flow rate, composition, and elution program were adopted on the basis of the conditions recommended in the technical data sheet of the instrument for the analysis of these compounds [41, 44]. Two different columns packed with the same stationary phase were tested: Phenomenex Luna Omega C_18_ (100 × 2.1 mm and 1.6 µm) and ACQUITY UPLC HSS T3 (100 × 2.1 mm and 1.8 µm). No significant differences in resolution efficiency were observed between both columns, so ACQUITY UPLC HSS T3 was selected as it provided lower system pressure, thus avoiding overpressure situations in the instrument.

Due to the higher number of NAs compared to those used in the technical data sheet, correct separation of all analytes was not achieved from the initial conditions tested for mobile phase composition (solvent A, 0.1% v/v formic acid aqueous; solvent B, methanol with 0.1% v/v formic acid), flow rate (0.5 mL min^−1^), and elution program. Special attention was paid to the separation of NDBA and NDiBA, as these compounds overlapped in these initial tests. It was therefore decided to carry out a test to see if changing the composition of the mobile phase would allow the separation of both compounds. Both solvents A and B of the mobile phase were modified by the addition of ammonium formate at 5 mM, resulting in excellent peak shape, but complete separation of NDBA and NDiBA was not achieved. Finally, after several experiences with different flow rates and elution programs, the best resolution among the 14 NAs was obtained with a mobile phase flow rate of 0.4 mL min^−1^, and the elution program was as follows: started at 5% B (held for 0.5 min), increased to 15% B at 1 min and held for 1 min, being then observed the elution of NDMA. It was then increased linearly to 65% B at 6 min, allowing the elution of NMOR, NEMA, NPYR, NDEA, and NPIP. This composition was maintained until 9 min, allowing the elution of NMPhA, NDPA, NEPhA, NDBA, and NDiBA with no overlap observed. This was then increased to 95% B until 10 min and maintained for 1 min, allowing the elution of NDPhA, NDBzA, and NDCHA. Finally, it was decreased to 5% B at 11.5 min and held for 3.5 min, giving a total analysis time of 15 min (Table 1).

### Evaluation of sample treatment

In order to optimize the sample treatment for efficient isolation of NAs, the UAE technique was selected. Initially, 5 mL of water was added to 0.5 g of sample (cooked ham or feces), and the mixture was sonicated for 5 min. For protein precipitation, 0.5 mL of 55% w/v TCA was then added, after which the mixture was centrifuged at 10 °C and 6000 rpm for 5 min. Finally, the supernatant was filtered, and the pH was adjusted to 3 with a NaOH solution to ensure compatibility with the chromatographic column.

To eliminate the need for this subsequent pH adjustment and simplify the process, a variant was evaluated in both matrices. In this variant, 5 mL of water was replaced with 5 mL of 5% v/v formic acid [45], and the use of TCA and the neutralization step with NaOH was omitted. No appreciable differences were observed when comparing both procedures (Fig. S1), so formic acid was selected as it offered a simpler alternative for sample preparation.

Different sample amounts were evaluated due to the complexity of both biological matrices. Three experiments were carried out with 0.1, 0.3, and 0.5 g of sample. For feces, 0.3 g of sample allowed to obtain higher sensitivities (Fig. S2). Increasing the amount of sample, the matrix effect became significant for most, resulting in a reduction of the analytical signal for several NAs. For cooked ham, the optimal sample amount was 0.1 g, as it allowed to obtain narrower and sharper chromatographic peaks. Additionally, filtering the extract from cooked ham samples larger than 0.1 g was difficult due to their content of insoluble compounds, so several filters had to be used.

### Method validation

The regression coefficients (*R*^2^) obtained for all analytes, in the absence and presence of the two evaluated matrices (cooked ham and feces), were greater than 0.99, demonstrating excellent linearity in the range of concentrations studied. In all cases, significant differences (*P*-value < 0.05) were observed between the slopes of the curves corresponding to the matrices and the aqueous standards, confirming the presence of a matrix effect preventing quantification of the samples against aqueous standards. Then, the two concentration levels (25 and 200 ng g^−1^, except for NDMA which was performed at 200 and 500 ng g^−1^) of each NA were evaluated in different types of ham and fecal samples. The results showed an RSD of less than 13.3% in all cases. This indicates good homogeneity between the samples within each matrix. Therefore, it was unnecessary to create a curve for each study group, as no significant differences in the NA signal were observed between the groups.

Next, 14 calibration curves were constructed, one for each analyte. The excellent linearity in the ranges studied was demonstrated by *R*^2^ values higher than 0.99 (Table S1). The linear ranges obtained for the fourteen calibration curves are shown in Table 2.

**Table 2:** Method validation data for the determination of NAs in cooked ham and feces samples

AnalyteAQ	Cooked ham samplesAQ		Feces samplesAQ AQ	
	Linear range, ng g−1AQ	LOD, ng g−1AQ	Linear range, ng g−1AQ	LOD, ng g−1AQ
NDMAAQ	47–1000AQ	14AQ	19–1000AQ	5.6AQ
NMORAQ	5.6–1000AQ	1.7AQ	3.8–1000AQ	1.2AQ
NEMAAQ	7.7–1000AQ	2.3AQ	1.8–1000AQ	0.54AQ
NPYRAQ	4.9–1000AQ	3.1AQ	5.3–1000AQ	1.6AQ
NDEAAQ	4.3–1000AQ	1.3AQ	2.2–1000AQ	0.67AQ
NPIPAQ	8.1–1000AQ	2.4AQ	1.6–1000AQ	0.48AQ
NMPhAAQ	4.8–1000AQ	1.4AQ	0.73–1000AQ	0.25AQ
NDPAAQ	4.3–1000AQ	1.3AQ	1.8–1000AQ	0.54AQ
NEPhAAQ	12–1000AQ	3.7AQ	3.9–1000AQ	1.2AQ
NDBAAQ	2.0–1000AQ	0.59AQ	0.44–1000AQ	0.13AQ
NDiBAAQ	2.4–1000AQ	0.63AQ	0.47–1000AQ	0.14AQ
NDPhAAQ	7.8–1000AQ	2.4AQ	2.7–1000AQ	0.82AQ
NDBzAAQ	24–1000AQ	7.1AQ	3.9–1000AQ	1.2AQ
NDCHAAQ	18–1000AQ	5.3AQ	7.1–1000AQ	2.1AQ

*LOD*, limit of detection

In addition, LODs ranging from 0.59 to 14 ng g^−1^ were found for the cooked ham matrix and from 0.13 to 5.6 ng g^−1^ for the feces matrix (Table 2). The most sensitive analyte was NDBA, and the least sensitive was NDMA in both types of samples.

In all cases, the results obtained in terms of RSD for the intraday and interday precision studies showed values of less than 8.8% and 12.4%, respectively. On the other hand, trueness, expressed as recovery, was calculated by dividing the measured concentration by the actual concentration and expressing the result as a percentage. The obtained values were found to be between 88 and 109%.

Stability studies of the standard solutions showed excellent results with RSD values between 1.2 and 4.3%, indicating that the solutions remain stable for at least 48 h. Regarding sample stability, on the other hand, it was observed that the signal varied considerably after 48 h for both matrices. Therefore, it was decided that the samples should be prepared on the same day of the analysis.

Finally, for carry-over studies, it was found that in no case did the signal of the blank vary with an RSD greater than 4.0%.

**Fig. 1:**
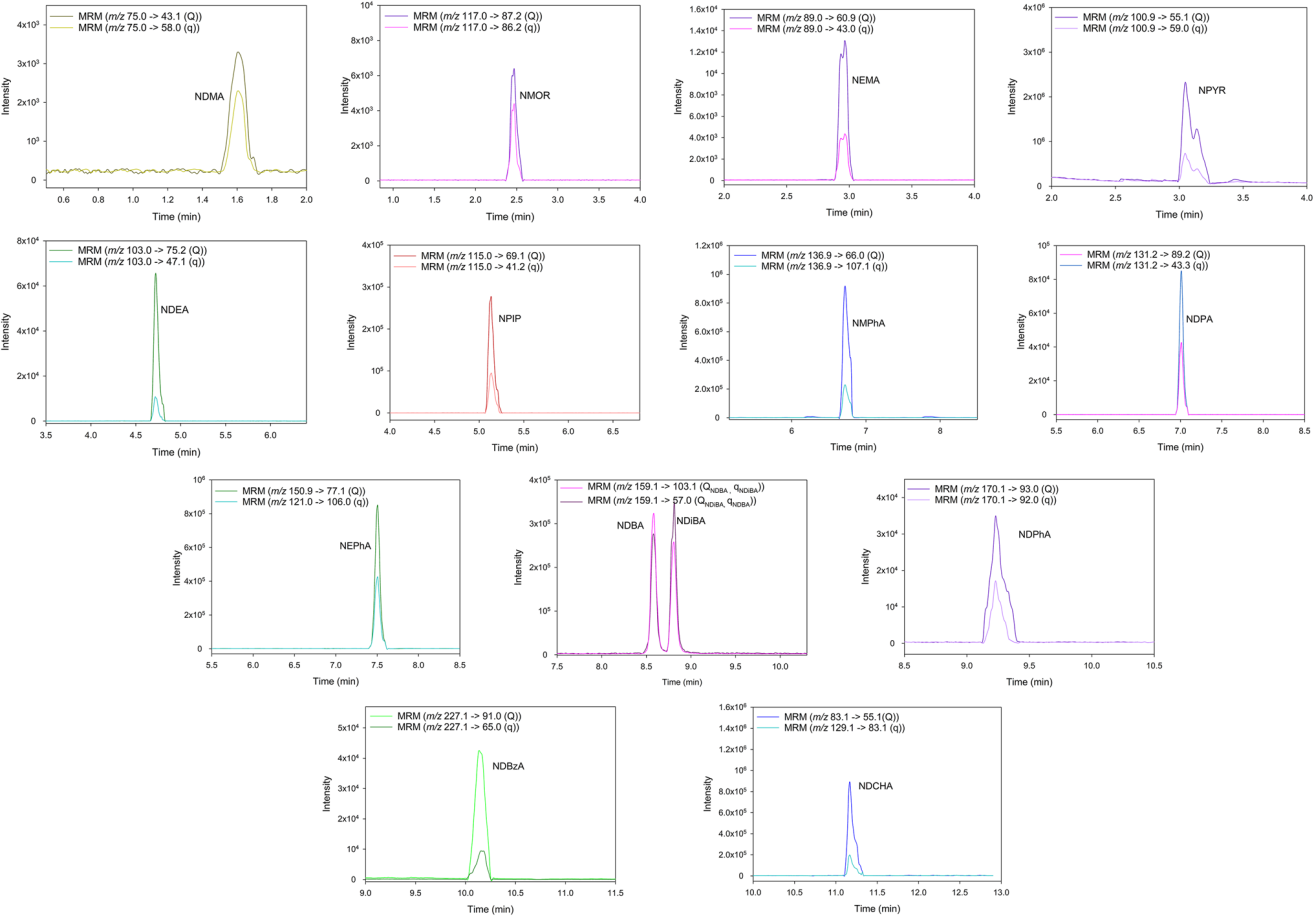
Chromatograms obtained for a fecal sample spiked with 100 ng g^−1^ NAs

The chromatograms obtained for feces sample spiked with 100 ng g^−1^ of all NAs using the proposed method are shown in Fig. 1. Similarly, Fig. S3 shows the chromatograms obtained for a cooked ham sample enriched with 100 ng g^−1^.

### Analysis of samples and statistical studies

The application of the proposed methodology allowed the evaluation of the NAs present in cooked ham and in fecal samples obtained from animals fed with different ham-based diets (without nitrites, with nitrites, and with polyphenols). The aim was to compare the concentrations detected in the different groups to see if there were any significant differences.

Table 3 summarizes the results obtained for the cooked ham samples. None of the samples contained detectable levels of NMOR, NEMA, NDEA, NDPA, NPIP, NEPhA, NDPhA, NDBzA, and NDCHA. The other five NAs, however, were sporadically detected across the sample set. NDMA was the most concentrated NA detected in both hams produced without nitrites and with nitrites. In contrast, the NA detected at the highest concentration in hams produced with polyphenols was NMPhA. Additionally, the total concentration of NAs detected in the cooked ham samples in this study aligns with previous studies on this product [21, 24].

**Table 3:** Concentration of NAs found in the cooked ham samples analyzed

GroupAQ	AnalyteAQ	Range, ng g−1AQ	Mean^b^ ± SD, ng g−1AQ	Incidence, %AQ	Median, ng g−1AQ
Nitrite-free hamAQ	NDMAAQ	ND (47)^c^–93AQ	22 ± 37AQ	30.0AQ	0.0AQ
	NPYRAQ	ND (3.1) –3.1AQ	0.63 ± 1.3AQ	20.0AQ	0.0AQ
	NMPhAAQ	ND (15)–48AQ	6.3 ± 15AQ	20.0AQ	0.0AQ
	NDBAAQ	ND (3.1) –20AQ	4.3 ± 5.9AQ	60.0AQ	3.4AQ
	NDiBAAQ	ND (2.4)–18AQ	6.8 ± 7.0AQ	70.0AQ	4.9AQ
	Total NAs^a^AQ	ND (2.4)–93AQ	40 ± 37AQ	90.0AQ	25AQ
Nitrite hamAQ	NDMAAQ	ND (49) –72AQ	10 ± 24AQ	16.7AQ	0.0AQ
	NPYRAQ	NDAQ	0AQ	0.0AQ	0.0AQ
	NMPhAAQ	ND (1.4)–34AQ	7.0 ± 13AQ	33.3AQ	0.0AQ
	NDBAAQ	ND (0.59)–2.9AQ	1.4 ± 1.3AQ	66.7AQ	2.1AQ
	NDiBAAQ	ND (0.63)–11AQ	4.8 ± 3.7AQ	75.0AQ	5.1AQ
	Total NAs ^a^AQ	ND (2.7)–107AQ	23 ± 33AQ	75.0AQ	9.0AQ
Ham with polyphenolsAQ	NDMAAQ	ND (14) –14AQ	3.5 ± 6.4AQ	25.0AQ	0.0AQ
	NPYRAQ	ND (3.1)–3.1AQ	0.26 ± 0.90AQ	8.33AQ	0.0AQ
	NMPhAAQ	ND (22)–64AQ	14 ± 24AQ	33.3AQ	0.0AQ
	NDBAAQ	ND (0.59)–3.2AQ	0.73 ± 0.95AQ	66.7AQ	0.98AQ
	NDiBAAQ	ND (3.6)–7.8AQ	3.4 ± 2.8AQ	66.7AQ	3.8AQ
	Total NAs ^a^AQ	ND (4.4)–88AQ	22 ± 30AQ	66.7AQ	6.6AQ

^a^Sum of the individual concentrations of NAs detected in each sample

^b^The mean was calculated by considering values below the LOD as zero and values detected between the LOD and LOQ as the LOD

^c^The value shown in parentheses is the minimum numerical value detected. *SD*, standard deviation; *ND*, not detected; *NQ*, not quantified

The results for the fecal samples were summarized in Table 4. NDMA, NMOR, NEMA, NPYR, NPIP, NDBzA, and NDCHA were below LODs in all cases. However, the other seven NAs were present in a subset of the samples, indicating a variable distribution. The NA detected at the highest levels was NDPhA, highlighting that while feces from animals fed diets rich in nitrites or in the absence of nitrites reached concentrations above 50 ng g^−1^, this NA was not detected in any of the fecal samples from rats fed diets rich in polyphenols. It should also be noted that in the two groups not fed nitrites, NDEA, one of the NAs classified by IARC as the most dangerous, was not detected, whereas in the group of rats fed a diet containing nitrites, NDEA was detected in 33.3% of the samples.

**Table 4:** Concentration of NAsAQ found in the feces samples analyzed

GroupAQ	AnalyteAQ	Range, ng g−1AQ	Mean^b^ ± SD, ng g−1AQ	Incidence, %AQ	Median, ng g−1AQ
Nitrite-free dietAQ	NDEAAQ	NDAQ	0.0AQ	0.0AQ	0.0AQ
	NDPAAQ	ND (0.54)^c^–0.54AQ	0.22 ± 0.28AQ	40.0AQ	0.0AQ
	NMPhAAQ	ND (2.5)–45AQ	8.6 ± 14AQ	60.0AQ	4.0AQ
	NEPhAAQ	ND (1.2)–3.9AQ	1.6 ± 2.0AQ	40.0AQ	0.0AQ
	NDBAAQ	ND (0.13)–3.6AQ	1.7 ± 1.3AQ	90.0AQ	1.3AQ
	NDiBAAQ	ND (0.14)–2.5AQ	0.81 ± 1.1AQ	50.0AQ	0.07AQ
	NDPhAAQ	ND (48)–52AQ	10 ± 21AQ	20.0AQ	0.0AQ
	Total NAs^a^AQ	0.13–63AQ	23 ± 23AQ	100AQ	15AQ
Nitrite dietAQ	NDEAAQ	ND (0.67)–5.0AQ	0.88 ± 1.8AQ	33.3AQ	0.0AQ
	NDPAAQ	NDAQ	0.0AQ	0.0AQ	0.0AQ
	NMPhAAQ	ND (1.6)–24AQ	5.7 ± 9.7AQ	41.7AQ	0.0AQ
	NEPhAAQ	ND (3.9)–21AQ	2.1 ± 6.0AQ	16.7AQ	0.0AQ
	NDBAAQ	NQ (0.13)–5.3AQ	2.1 ± 1.5AQ	100AQ	1.9AQ
	NDiBAAQ	ND (0.48)–6.0AQ	1.1 ± 2.0AQ	41.7AQ	0.0AQ
	NDPhAAQ	ND (38)–57AQ	25 ± 27AQ	50.0AQ	19AQ
	Total NAs^a^AQ	5.3–100AQ	37 ± 32AQ	100AQ	31AQ
Diet with polyphenolsAQ	NDEAAQ	NDAQ	0.0AQ	0.0AQ	0.0AQ
	NDPAAQ	NDAQ	0.0AQ	0.0AQ	0.0AQ
	NMPhAAQ	ND (1.5)–4.8AQ	0.52 ± 1.4AQ	16.7AQ	0.0AQ
	NEPhAAQ	ND (1.2)–3.9AQ	0.98 ± 1.8AQ	25.0AQ	0.0AQ
	NDBAAQ	ND (1.1)–3.5AQ	1.3 ± 1.4AQ	58.3AQ	1.3AQ
	NDiBAAQ	ND (0.14)–3.1AQ	0.78 ± 1.3AQ	41.7AQ	0.0AQ
	NDPhAAQ	NDAQ	0.0AQ	0.0AQ	0.0AQ
	Total NAs^a^AQ	ND (0.14)–13AQ	3.6 ± 3.6AQ	83.3AQ	2.6AQ

^a^Sum of the individual concentrations of NAs detected in each sample

^b^The mean was calculated by considering values below the LOD as zero and values detected between the LOD and LOQ as the LOD

^c^The value shown in parentheses is the minimum numerical value detected. *SD*, standard deviation; *ND*, not detected; *NQ*, not quantified

**Fig. 2:**
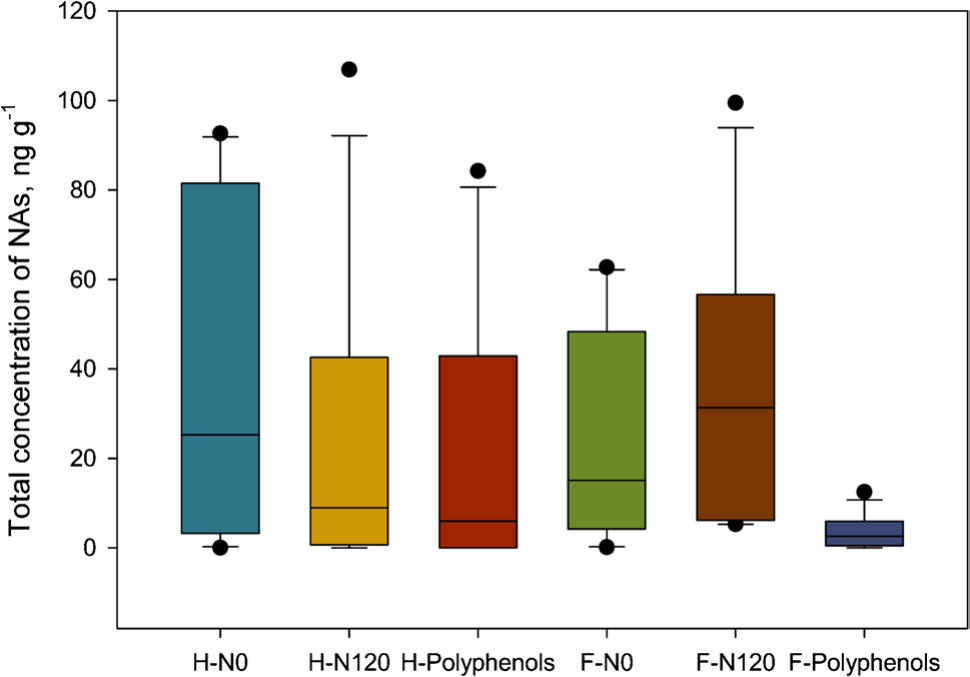
Total nitrosamine concentration (mean ± standard deviation) in ham (H) and feces (F), categorized by the type of preservative used: free of preservatives (N0), nitrite (N120), or polyphenols

To show the differences in total NA concentrations depending on the matrix type and preservative used, the mean values per group were plotted alongside their standard deviation (Fig. 2). At first glance, it can be seen that the fecal samples from rats fed a polyphenol diet showed a significantly lower total NA concentration compared to the other study groups. In contrast, no notable differences were observed among the three study groups in the cooked ham samples due to the large error bars, indicating high variability.

To evaluate the joint effect of preservative type (nitrite-free, nitrites or polyphenols) and matrix (ham or feces) on the NAs concentration profile, a two-way PERMANOVA analysis was performed. The model was estimated using 9999 permutations to ensure that the significance values were estimated robustly. The results indicated a statistically significant effect of matrix type (*P*-value = 0.0001), suggesting marked differences in NA profiles between ham and feces. In contrast, the main effect of the preservative was not significant (*P*-value = 0.0568). However, the *P*-value was very close to the conventional threshold of 0.05, suggesting a possible tendency to generate differences that deserves further exploration. Furthermore, a significant interaction was detected between preservative type and matrix (*P*-value = 0.006), indicating that the preservative’s effect varies according to the analyzed matrix. Taken together, these results show that NA profiles mainly vary according to the matrix (cooked ham or feces) and that the impact of the preservative used differs between the two matrices. These results suggest that biological processes occurring after ingestion of food can modify NA formation or elimination, which is important when evaluating NA risk to the organism. Therefore, independent analyses by sample type are required to study more precisely the effect of the preservative used.

To evaluate the effect of the type of preservative used on the concentration profile of NAs detected in each matrix independently, a one-way PERMANOVA was performed. For cooked ham, this analysis revealed no significant differences between the preservatives used (*P*-value = 0.3122). Pairwise comparisons between groups (free nitrite, nitrite, and polyphenols) also revealed no statistically significant differences (*P*-values > 0.13, Table S2), indicating that using different preservatives does not significantly alter the NA profile in the analyzed meat products. In contrast, the type of preservative did have a significant effect on the NA concentration profile in the fecal matrix (*P*-value = 0.008). Pairwise comparisons (Table S3) revealed significant differences between the nitrite-treated group and polyphenol-treated group (*P*-value = 0.0027), as well as between the no preservative group and the polyphenol-treated group (*P*-value = 0.0022). However, the difference between the no preservative group and nitrite-treated group was more subtle (*P*-value = 0.160).

These results suggest that preservatives do not significantly influence the NA profile on cooked ham. This finding can be attributed to the production process, as the formation of NAs in meat products is primarily associated with the use of nitrites at temperatures above 100 °C. Numerous studies confirm that processing meat products containing nitrites at high temperatures, such as during roasting or frying, produces large amounts of N-nitrosamines [4]. In our study, the manufacturing process for cooked hams involves cooking at a lower temperature (68 °C), which inhibits the formation of N-nitrosamines, even in the group treated with nitrites. This is the main reason for the absence of differences in NA levels between groups of cooked hams. However, they do influence the modulation of the NA profile after passage through the digestive system, as evidenced by the differences detected in fecal samples. This indicates a possible effect of preservatives on the formation, transformation, or availability of NAs at the intestinal level, which could have important toxicological implications.

Since differences were observed among the groups in the fecal matrix, a more in-depth analysis was conducted by performing individual comparisons for each NA in the feces. A Kruskal–Wallis test was applied to each NA since the data did not follow a normal distribution. Significant differences were found in the concentration of NDEA (*P*-value = 0.018), NDPA (*P*-value = 0.005), and for NDPhA (*P*-value = 0.014). A Kruskal–Wallis test was then performed again, this time on the total level of NAs per sample, and significant differences between the study groups were observed (*P*-value = 0.002). Dunn’s test was then performed, which showed that there were significant differences in the total concentration of NAs between the feces of animals fed the nitrite diet and those fed the polyphenol-rich diet. Correlating these results with the data presented in Table 4, it can be concluded that the presence of NAs is higher in nitrite diet-fed animals with a mean total NAs concentration of 37 ng g^−1^ and with all samples being positive for one or more of the 7 NAs detected in the fecal samples. In contrast, the total concentration of NAs is significantly lower in rats fed a polyphenol-rich diet, with a mean total concentration of 3.6 ng g^−1^ and two samples not testing positive for any NAs.

These findings confirm that, although the use of preservatives does not significantly alter the NA profile in ham, it does influence the presence of specific compounds, such as NDEA, NDPA, and NDPhA, after the digestive process. Notably, the use of nitrite as a preservative results in a greater difference compared to the use of polyphenols, suggesting a possible protective effect of the latter against the formation of NAs in the organism. Furthermore, previous research studying the same animal model where colon cancer lesions were induced with the diet indicates that a polyphenol diet also reduces the formation of genotoxic fecal aldehydes and preneoplastic lesions [20]. Therefore, both studies agree that the addition of polyphenols to the diet reduces the production of toxic fecal metabolites.

**Fig. 3:**
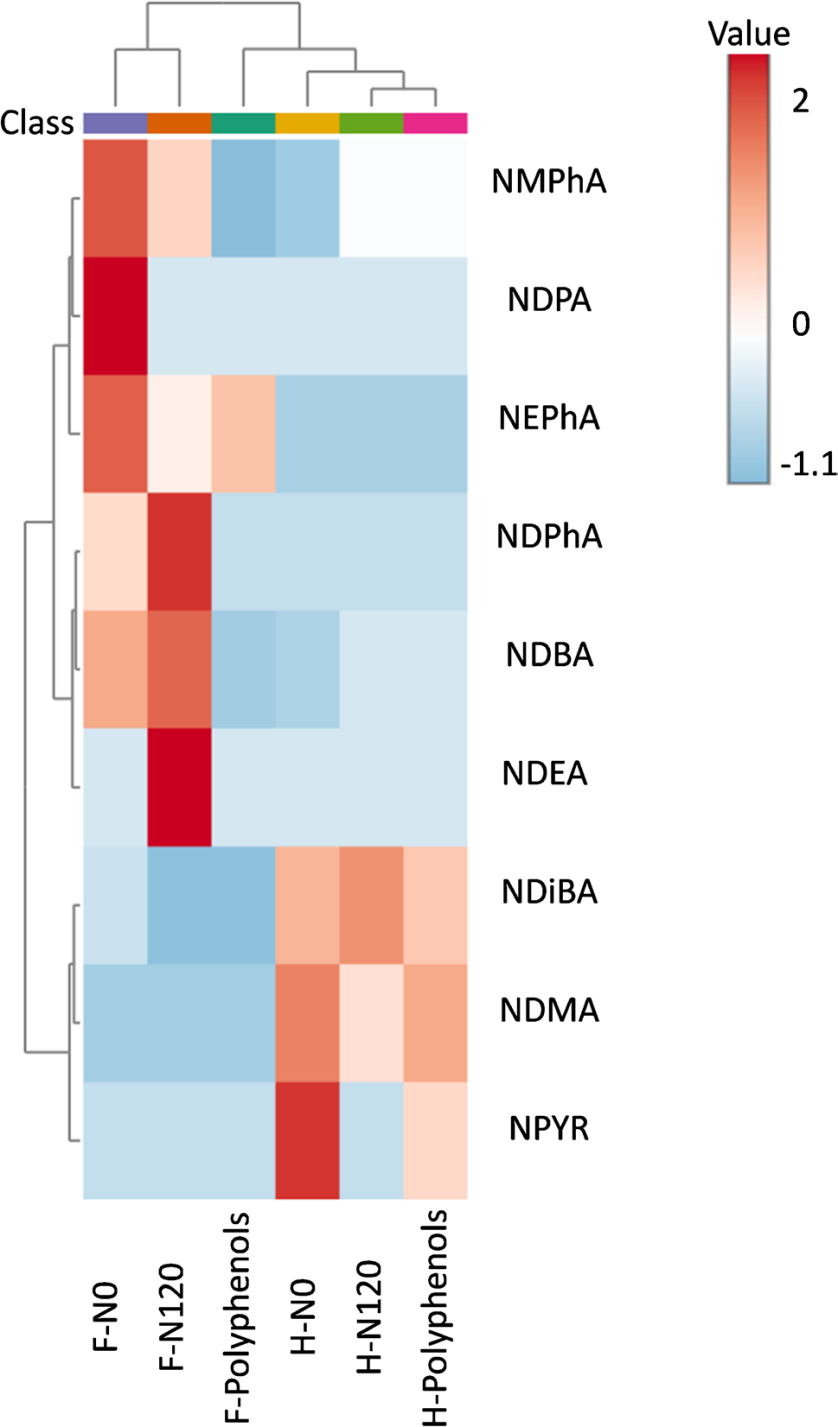
Heatmap showing the distribution of the presence of nitrosamines according to the type of matrix (ham (H) and feces (F)) and the type of preservative (free of preservatives (N0), nitrite (N120) or polyphenols)

To illustrate the distribution of each NA under the different study conditions, a heatmap (Fig. 3) was created showing the presence or absence of each NA in each sample, according to the matrix and type of preservative used. This visualization enables the quick identification of compounds that appear in one matrix or both, and the experimental conditions under which they appear. Thus, cases such as NDMA (present only in cooked ham) and NEPhA and NDPhA (present only in feces) stand out.

Finally, the possible association between the presence of NAs in both the cooked ham and the animal’s feces was evaluated to determine whether these compounds are present in both or only in one or neither. This approach enabled compounds that may be associated with transformations occurring during the digestive process to be identified. For NAs detected in both matrices, 2 × 2 contingency tables were constructed to code cases where the compound was present in both, one or neither of the matrices. Fisher’s exact test was applied. The results are summarized in Table S4. Statistically significant differences were detected for NMPhA (*P*-value = 0.002) and NDiBA (*P*-value < 0.001), indicating that these compounds are not randomly distributed between the matrices but are associated with one another.

In the case of NMPhA, the detections in both matrices were frequent, whereas individual detections in only one matrix were less common. This pattern suggests that when NMPhA is present in cooked ham, it is more likely to be found in the animal feces that were fed that ham. This indicates a possible relationship between the presence of NMPhA in a diet and its subsequent appearance in the organism. NDBA was detected simultaneously in both matrices in 17 cases, making it the most frequent combination in the contingency matrix. While this coincidence was notable, Fisher’s exact test yielded a *P*-value of 0.116, indicating that the association was not statistically significant. Nevertheless, the observed trend may indicate a relationship worth exploring with a larger sample. In the case of NDiBA, the pattern was particularly clear: it was never detected in feces unless it was also present in ham. This unidirectional dependence suggests that presence in the meat matrix is a prerequisite for its appearance in feces. Fisher’s test confirmed this strong association, yielding a *P*-value of less than 0.001. Taken together, these results reinforce the hypothesis that some NAs remain stable throughout the digestive process, reflecting their presence in the fecal matrix after being ingested. Specifically, NMPhA exhibited significantly different levels in the nitrite group (*P*-value = 0.010), suggesting a potential interaction between this preservative and the bioavailability or stability of these molecules during digestion.

On the other hand, a *Z*-test for comparison of proportions was applied to NAs that were detected in only one of the matrices and generated empty cells in the Fisher matrix. The results are presented in Table S5. Significant differences were found overall for NDMA (*P*-value = 0.005), NDEA (*P*-value = 0.039), NEPhA (*P*-value = 0.005), and NDPhA (*P*-value = 0.003). This differential pattern could be related to specific transformation or formation processes during digestion, or to the stability of each NA depending on the chemical environment of each matrix. In the case of NDMA, which was detected exclusively in ham, it is possible that it forms during the thermal processing or curing of the product, but that it is unstable during digestion or rapidly degraded and not excreted in detectable amounts. Conversely, the exclusive presence of NDEA, NEPhA, and NDPhA in feces suggests that these NAs may be generated in situ during digestion. Taken together, these results support the idea that certain NAs are distributed non-randomly between matrices, depending on the type of preservative used.

Finally, comparison of the analytical method developed in this study with other previously published methods (Table 5) reveals several significant advantages. First, it enables the simultaneous determination of a greater number of NAs (14 in total). Additionally, it has been developed to analyze both cooked ham and fecal samples, offering the possibility of jointly evaluating daily exposure and the presence of these compounds in biological matrices. Furthermore, sample preparation is simpler than in most protocols, requiring less time (15 min) and a minimal amount of solvent (5 mL). Finally, this method offers a novel perspective on food safety by analyzing how different additives, such as polyphenols and nitrites, affect NA formation in meat products and feces. The latter has been a relatively unexplored area until now.

**Table 5:** Comparison of different methods for nitrosamines analysis

Sample	Nº NAs		Instrument	Sample preparation	Objetive	Ref
Sauges		6	GC-MS (24 min)	HF-EME-SLM Time: 50 min; high amount of solvent: > 50 mL	Evaluate the concentration of NAs in meat products and their potential health risk	[17]
Hams		7	GC-MS (14 min)	UAE Time: > 50 min; high amount of solvent: > 80 mL	Evaluate the concentration of NAs in meat products and their potential health risk	[21]
Sauges		6	GC-MS (24 min)	UAE + HF-EME Time: > 45 min; high amount of solvent: > 50 mL	Evaluate the concentration of NAs in meat products and their potential health risk	[22]
Processed beef		6	GC-MS (16 min)	Multiples extraction Time: > 60 min; high amount of solvent: > 100 mL	Evaluate the concentration of NAs in meat products and their potential health risk	[23]
Human feces		7	GC-TEA (21 min)	Distillation + LLE Time: > 75 min; High amount of solvent: > 80 mL	Evaluate the concentration of NAs in feces	[25]
Biological samples		8	GC-TEA (21 min)	Distillation + Extraction Time: not specified; high amount of solvent: > 85 mL	Evaluate the presence of nitrite compounds in biological samples	[26]
Rat feces		8	GC-MS (28 min)	Multiples SPE Time: > 60 min; high amount of solvent: > 50 mL	Evaluate the concentration of NAs in feces	[27]
Red meat		9	GC–MS (31 min)	HS-SPME Time: 35 min; low amount of solvent: 10 mL	Evaluate the concentration of NAs in meat and their potential health risk	[34]
Meat and fish		11	LC-MS/MS (15 min)	QuEChERS Time: > 60 min; medium amount of solvent: 20 mL	Evaluate the concentration of NAs in fish and meat products	[36]
Cooked ham and rat feces		14	LC-MS/MS (15 min)	UAE Time: 15 min; low amount of solvent: 5 mL	Evaluate the concentration of NAs in cooked ham and feces of rats	This work

*HF-EME-SLM*, hollor fiber-electromembrane extraction-supported liquid membrane; *UAE*, ultrasound-assisted extraction; *LLE*, liquid–liquid extraction; *SPE*, solid phase extraction; *HS-SPME*, headspace-solid phase microextraction

### Evaluation of the greenness and innovation of the proposed method

**Fig. 4:**
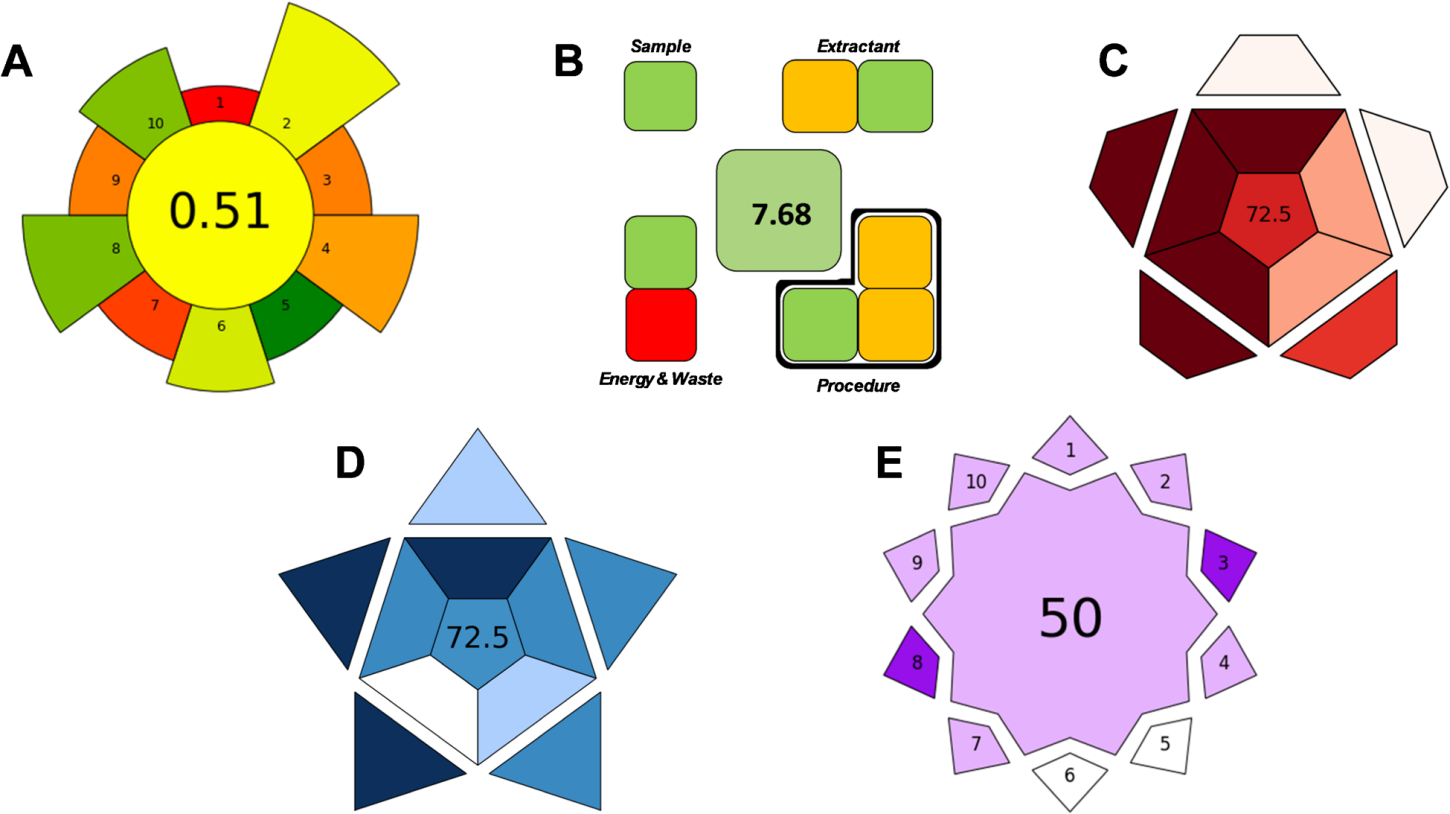
Pictograms illustrating the results of the greenness assessment obtained using: **A** AGREEprep, **B** Sample Preparation Metric Of Sustainability (SPMS), **C** Red Analytical Performance Index (RAPI), **D** Blue Applicability Grade Index (BAGI), and **E** Violet Innovation Grade Index

Several metric tools have recently been developed to assess environmental sustainability, efficiency, and the innovative nature of analytical procedures. AGREEprep is designed to analyze the sustainability of sample preparation stages using the ten principles of green sample preparation (GSP) as a reference. This tool uses a scale from 0, indicating no sustainability, to 1, indicating maximum sustainability (totally green) [46]. The method proposed in this study obtained a score of 0.51 in AGREEprep (Fig. 4A), reflecting an intermediate level of sustainability. Among the positive aspects of this procedure are the low quantity of samples used, moderate energy consumption, the reduced number of risks associated with the procedure, and the relatively low volume of acid used (0.25 mL in 4.75 mL of water). Negative aspects identified include the need to perform the procedure ex situ (in the laboratory), the use of a large number of non-reusable materials and reagents, and the lack of automation. Overall, these results demonstrate that, despite several areas for improvement, the procedure can be considered to have acceptable environmental performance.

Another tool used to evaluate the procedure was the Sample Preparation Metric Of Sustainability (SPMS), which evaluates the sample preparation stage exclusively and explicitly in terms of sustainability [47]. After evaluating the nine parameters considered, a final score of 7.68 out of 10 (Fig. 4B) was obtained, indicating favorable performance. The favorable evaluation was influenced by several factors, including the small sample amount used, the type of acid used for extraction, and the absence of evaporation, dilution, or derivatization stages. However, the need for cooling during centrifugation was identified as a negative aspect, as this slightly affected the evaluation. Other negative aspects were the use of an acid as extractant and the need for more than one manual step to carry out the described procedure. In summary, this score indicates that the procedure is sustainable and efficient, with scope for minor improvements.

The Red Analytical Performance Index (RAPI) and the Blue Applicability Grade Index (BAGI) were also evaluated. These are two complementary tools used to measure the practical applicability of the proposed analytical method [48]. Ten different parameters were evaluated in each tool to obtain the score for both indices. In both cases, a total score of 72.5 out of 100 was obtained, reflecting the high applicability of the method (Fig. 4C and D). When applying RAPI, it can be seen that the method provides excellent validation results in terms of accuracy, linearity, the width of the studied calibration range, and robustness. However, the lack of studies related to possible interferents slightly hinders it, despite the highly sensitive instrumental technique used, as does the low average concentration of the samples, which, in the case of some NAs, is close to the LOQ. Regarding BAGI, the proposed method is straightforward as it uses commonly available reagents and does not require complex preconcentration steps. However, it is hampered by the analytical instrumentation used, as this equipment is not commonly found in routine laboratories.

Finally, the Violet Innovation Grade Index (VIGI), a metric designed to evaluate the degree of innovation of analytical methods, was studied [49]. In this research, a score of 50 out of 100 was obtained by evaluating ten innovation-related criteria (Fig. 4E). This score indicates an intermediate level of innovation, reflecting the fact that the method incorporates certain novel elements, such as the analytical instrumentation used, its applicability to various areas of knowledge, and the relevance of the issue addressed. However, the method’s degree of innovation is limited by the use of non-innovative reagents and materials, as well as the lack of miniaturization stages. In this sense, the procedure can be considered competitive in terms of innovation, although there is room for improvement to achieve higher values.

Overall, evaluation of the proposed method using different metrics showed that it achieves acceptable and efficient environmental performance and has a high degree of applicability and intermediate innovation.

## Conclusions

The UAE technique for sample treatment and HPLC-APCI with QqQ-MS/MS analysis was found to be suitable for the quantification of 14 NAs in cooked ham and fecal samples from animals fed with these cooked hams. This technique allows efficient isolation of the analytes by a very fast and simple procedure, as well as their unequivocal detection and quantification. Thus, high sensitivity was achieved, with LOQs in the order of ng g^−1^ for all analytes in both matrices, together with high levels of precision and accuracy. Furthermore, the application of this methodology enabled the evaluation of the effect of incorporating polyphenols into cooked ham on NA levels in feces for the first time, offering a novel and highly interesting approach to studying the formation of these compounds. The procedure was also evaluated in terms of sustainability, efficiency, and innovation, obtaining favorable scores for the different metric tools used (AGREEprep, SPMS, RAPI, BAGI, and VIGI). Analysis of the samples revealed the presence of quantifiable levels of five NAs in the cooked ham and seven NAs in the feces. Multivariate tests revealed that the concentration profile of NAs primarily depends on the biological matrix (cooked ham or feces). When analyzing the two matrices separately, it was observed that preservatives modulate the NA profile after digestion. Specifically, significantly lower levels were found in animals fed a polyphenol-rich cooked ham diet than in those fed nitrite. These results suggest that incorporating polyphenols into cooked ham-based diets as a nitrite substitute significantly reduces the concentration of NAs detected in the feces of the in vivo animal model. Furthermore, the detection of NDMA only in cooked ham and of NDEA, NEPhA, and NDPhA only in feces suggests that certain NAs are generated, transformed, or degraded during digestion. Taken together, these findings support the hypothesis of a non-random distribution of NAs between matrices and highlight the significant role of preservative type in the formation or stability of these compounds at the intestinal level.

## Supplementary Information

Below is the link to the electronic supplementary material.

Supplementary Material 1 Fig. S1 Evaluation of the acidic medium used during the ultrasound-assisted extraction step on the sensitivity of NAs. Fig. S2 Evaluation of the fecal sample amount on the peak intensity of quantitative ion of each NA using ultrasound-assisted extraction. Fig. S3 Chromatograms obtained for a cooked ham sample spiked with 100 ng g−1 NAs. Table S1. Calibration curves for the determination of NAs in cooked ham and feces samples. Table S2. *P-value* of the PERMANOVA test in the cooked ham matrix. Table S3. *P-value* of the PERMANOVA test in the fecal matrix. Table S4. *P-value* of Fisher exact test to evaluate the association between the presence of NAs in cooked ham and in feces. Table S5. *P-value* of Z-test to compare the detection rate of nitrosamines specific to a matrix (cooked ham or feces) (PDF 478 KB)

## Data Availability

Data will be made available upon request.

## Declarations

### Source of biological material

Rats were provided by Charles River Laboratories (Saint-Germain-sur-l’Arbresle, France).

## References

[1] Choi NR, Kim YP, Ji WH, Hwang GS, Ahn YG. Identification and quantification of seven volatile n-nitrosamines in cosmetics using gas chromatography/chemical ionization-mass spectrometry coupled with head space-solid phase microextraction. Talanta. 2016;148:69–74. 10.1016/j.talanta.2015.10.045. 26653425 10.1016/j.talanta.2015.10.045

[2] Xie Y, Geng Y, Yao J, Ji J, Chen F, Xiao J, et al. N-nitrosamines in processed meats: exposure, formation and mitigation strategies. J Agric Food Res. 2023;13: 100645. 10.1016/j.jafr.2023.100645.

[3] International Agency for Research on Cancer. Agents classified by the IARC monographs. 2017. https://monographs.iarc.who.int/list-of-classifications. Accessed 14 July 2025.

[4] Schrenk D, Bignami M, Bodin L, Chipman JK, del Mazo J, Hogstrand C, et al. Risk assessment of N-nitrosamines in food. EFSA J. 2023;21:7884. 10.2903/j.efsa.2023.7884 PMC10043641 36999063

[5] Eisenbrand G, Buettner A, Diel P, Epe B, Först P, Grune T, et al. Commentary of the SKLM to the EFSA opinion on risk assessment of N-nitrosamines in food. Arch Toxicol. 2024;98:1573–80. 10.1007/s00204-024-03726-1. 38573336 10.1007/s00204-024-03726-1 PMC11106120

[6] Bonifacie A, Aubry L, Sayd T, Bourillon S, Duval A, Kombolo M, et al. Chemical effects of nitrite reduction during digestion of cured cooked and recooked meat on nitrosation, nitrosylation and oxidation. Food Res Int. 2024;195: 114969. 10.1016/j.foodres.2024.114969. 39277238 10.1016/j.foodres.2024.114969

[7] Lee IK, Park NY, Park SY, Jeong JH, Lee J, Moon B, et al. Assessment of nitrosamine exposure in Korean foods: analysis, risk evaluation, and implications. Food Sci Biotechnol. 2024;33:2417–26. 10.1007/s10068-024-01651-8. 39145132 10.1007/s10068-024-01651-8 PMC11319691

[8] Fan CC, Lin TF. N-nitrosamines in drinking water and beer: detection and risk assessment. Chemosphere. 2018;200:48–56. 10.1016/j.chemosphere.2018.02.025. 29475028 10.1016/j.chemosphere.2018.02.025

[9] Meikopoulos T, Begou O, Panagoulis T, Kontogiannidou E, Fatouros DG, Miller JH, et al. Uhplc-ms/ms method for the simultaneous determination of nicotine and tobacco-specific nitrosamines NNN and NNK for use in preclinical studies. Anal Bioanal Chem. 2022;414:7865–75. 10.1007/s00216-022-04319-6. 36163593 10.1007/s00216-022-04319-6 PMC9568479

[10] Alhooshani K. Determination of nitrosamines in skin care cosmetics using Ce-SBA-15 based stir bar-supported micro-solid-phase extraction coupled with gas chromatography mass spectrometry. Arab J Chem. 2020;13:2508–16. 10.1016/j.arabjc.2018.06.004.

[11] Giménez-Campillo C, Pastor-Belda M, Campillo N, Hernández-Córdoba M, Viñas P. Development of a new methodology for the determination of N-nitrosamines impurities in ranitidine pharmaceuticals using microextraction and gas chromatography-mass spectrometry. Talanta. 2021;223: 121659. 10.1016/j.talanta.2020.121659. 33298254 10.1016/j.talanta.2020.121659

[12] Pu C, Cavarra BR, Zeng T. Combining high-resolution mass spectrometry and chemiluminescence analysis to characterize the composition and fate of total N-nitrosamines in wastewater treatment plants. Environ Sci Technol. 2024;58:17081–91. 10.1021/acs.est.4c06555. 39254226 10.1021/acs.est.4c06555 PMC11428135

[13] Zhou Y, Zhai S, Yao G, Li J, Li Z, Ma Z, et al. Formation and prediction of heterocyclic amines and N-nitrosamines in smoked sausages using back propagation artificial neural network. J Sci Food Agric. 2024;104:4083–96. 10.1002/jsfa.13291. 38323696 10.1002/jsfa.13291

[14] Duan L, Wang C, Li Y, Yang B, Zheng X, Liu J, et al. Sensitive determination of volatile nitrosamines with ambient pressure ammonium-adduct ionization mass spectrometry. Anal Bioanal Chem. 2024;416:6839–47. 10.1007/s00216-024-05580-7. 39400577 10.1007/s00216-024-05580-7

[15] Ruiz-Saavedra S, Pietilä TK, Zapico A, de los Reyes-Gavilán CG, Pajari AM, González S. Dietary nitrosamines from processed meat intake as drivers of the fecal excretion of nitrosocompounds. J Agric Food Chem. 2024;72:17588–98. 10.1021/acs.jafc.4c05751. 39072357 10.1021/acs.jafc.4c05751 PMC11311235

[16] Sorour M, Mehanni A-H, Mahmoud E-S, El-Hawashy R. Nitrate, nitrite and N-nitrosamine in meat products. J Sohag Agriscice (JSAS). 2023;8:121–35. 10.21608/jsasj.2023.316218.

[17] Nabizadeh S, Aeini K, Barzegar F, Arabameri M, Hosseini H, Kamankesh M, et al. Volatile N-nitrosamines in processed meat products: an approach for monitoring dietary exposure, assessing human risk, and evaluating variable correlations by principal component analysis and heat map. Food Chem Toxicol. 2024;188: 114649. 10.1016/j.fct.2024.114649. 38599275 10.1016/j.fct.2024.114649

[18] Crews C. The determination of N-nitrosamines in food. Qual Assur Saf Crops Foods. 2010;2:2–12. 10.1111/j.1757-837X.2010.00049.x.

[19] Zhu J, Lu Y, He Q. From detection methods to risk prevention: control of N-nitrosamines in foods and the role of natural bioactive compounds. Compr Rev Food Sci Food Saf. 2024;23:e70000. 10.1111/1541-4337.70000. 39217507 10.1111/1541-4337.70000

[20] Giménez-Campillo C, Campillo N, Arroyo-Manzanares N, Martínez CM, de Torre-Minguela C, Viñas P. Metabolomics study of the formation of genotoxic molecules based on the fecal volatile metabolites profile using an in vivo animal model. Microchem J. 2024;199: 110132. 10.1016/j.microc.2024.110132.

[21] Özbay S. Investigation of volatile N-nitrosamine contents of hams with different ingredients produced in Turkey. J Food Compos Anal. 2024;130: 106170. 10.1016/j.jfca.2024.106170.

[22] Nabizadeh S, Barzegar F, Babaei M, Kamankesh M, Mohammadi A. New and efficient direct-SLM two-phase hollow fiber electromembrane extraction coupled to GC/MS for the analysis of nitrosamines in different types of sausage: investigation of meat type, meat percent and cooking methods. Food Chem. 2023;416: 135759. 10.1016/j.foodchem.2023.135759. 36893642 10.1016/j.foodchem.2023.135759

[23] Abdullah ATM, Khan TA, Sharif M, Mazumdar RM, Rahman MM. Determination of dietary exposure and extraction efficiency of nitrosamine from cooked meat. Curr Res Food Sci. 2022;5:491–7. 10.1016/j.crfs.2022.02.010. 35265857 10.1016/j.crfs.2022.02.010 PMC8898760

[24] Giménez-Campillo C, Pastor-Belda M, Campillo N, Hernández JDED, Guillén I, Vizcaíno P, et al. Ultrasound assisted extraction approach to test the effect of elastic rubber nettings on the N-nitrosamines content of ham meat samples. Foods. 2021;10: 2564. 10.3390/foods10112564. 34828845 10.3390/foods10112564 PMC8618317

[25] Lee LJ, Archer MC, Bruce WR. Absence of volatile nitrosamines in human feces. Cancer Res. 1981;41:3992–4. 7285009

[26] Tricker AR, Pfundstein B, Kalble T, Preussmann R. Secondary amine precursors to nitrosamines in human saliva, gastric juice, blood, urine and faeces. Carcinogenesis. 1992;13:563–8. 10.1093/carcin/13.4.563. 1576707 10.1093/carcin/13.4.563

[27] Zhao ZX, Chen SZ, Xia ZL, Bin Xu Y, Zhang LL, Tian SM, et al. High level nitrosamines in rat faeces with colorectal cancer determined by a sensitive GC-MS method. J Pharm Biomed Anal. 2022;210: 114576. 10.1016/j.jpba.2021.114576. 34998074 10.1016/j.jpba.2021.114576

[28] Sheweita SA, El Banna YY, Balbaa M, Abdullah IA, Hassan HE. N-nitrosamines induced infertility and hepatotoxicity in male rabbits. Environ Toxicol. 2017;32:2212–20. 10.1002/tox.22436. 28573719 10.1002/tox.22436

[29] Liu W, Huang J, Yan Z, Lin Y, Huang G, Chen X, et al. Association of N-nitrosodimethylamine exposure with cognitive impairment based on the clues of mice and humans. Front Aging Neurosci. 2023;15: 1137164. 10.3389/fnagi.2023.1137164. 37441677 10.3389/fnagi.2023.1137164 PMC10333700

[30] Wang H, Guo L, Liu F, Fan W, Chai G, Shi Q, et al. Brain distribution and metabolic profiling of 4-(methylnitrosamino)-1-(3-pyridyl)-1-butanone in rats investigated by UHPLC-HRMS/MS following peripheral administration. Anal Bioanal Chem. 2023;415:2317–27. 10.1007/s00216-023-04655-1. 37004550 10.1007/s00216-023-04655-1

[31] Domínguez-Oliva A, Hernández-Ávalos I, Martínez-Burnes J, Olmos-Hernández A, Verduzco-Mendoza A, Mota-Rojas D. The importance of animal models in biomedical research: current insights and applications. Animals. 2023;13: 1223. 10.3390/ani13071223. 37048478 10.3390/ani13071223 PMC10093480

[32] Wang X, Hu Y, Zhu W, Wang D. Investigation of metabolite alterations in the kidneys of methionine-choline-deficient mouse by mass spectrometry imaging. Anal Bioanal Chem. 2024;416:1011–22. 10.1007/s00216-023-05091-x. 38108841 10.1007/s00216-023-05091-x

[33] Chang SH, Ho HY, Zang CZ, Hsu YH, Lin MC, Tseng SH, et al. Screening of nitrosamine impurities in sartan pharmaceuticals by gc-ms/ms. Mass Spectrom Lett. 2021;12:31–40. 10.5478/MSL.2021.12.2.31.

[34] Sun C, Wang R, Wang T, Li Q. Primary evaluation of nine volatile N-nitrosamines in raw red meat from Tianjin, China, by HS-SPME-GC–MS. Food Chem. 2020;310: 125945. 10.1016/j.foodchem.2019.125945. 31837529 10.1016/j.foodchem.2019.125945

[35] Witkowska AB, Giebułtowicz J, Dąbrowska M, Stolarczyk EU. Development of a sensitive screening method for simultaneous determination of nine genotoxic nitrosamines in active pharmaceutical ingredients by GC-MS. Int J Mol Sci. 2022;23: 12125. 10.3390/ijms232012125. 36292981 10.3390/ijms232012125 PMC9603764

[36] Lee HJ, Jung YS, Seo D, Kim E, Yoo M. Development and validation of QuEChERS-based LC-MS/MS method for simultaneous quantification of eleven N-nitrosamines in processed fish meat, processed meat, and salted fish products. Food Chem. 2024;459: 140281. 10.1016/j.foodchem.2024.140281. 39047543 10.1016/j.foodchem.2024.140281

[37] Vogel M, Norwig J. Analysis of genotoxic N-nitrosamines in active pharmaceutical ingredients and market authorized products in low abundance by means of liquid chromatography – tandem mass spectrometry. J Pharm Biomed Anal. 2022;219: 114910. 10.1016/j.jpba.2022.114910. 35779354 10.1016/j.jpba.2022.114910

[38] Engemann A, Focke C, Humpf HU. Intestinal formation of N-nitroso compounds in the pig cecum model. J Agric Food Chem. 2013;61:998–1005. 10.1021/jf305040e. 23297847 10.1021/jf305040e

[39] Banerjee S. Empowering clinical diagnostics with mass spectrometry. ACS Omega. 2020;5:2041–8. 10.1021/acsomega.9b03764. 32064364 10.1021/acsomega.9b03764 PMC7016904

[40] Santarelli RL, Vendeuvre JL, Naud N, Taché S, Guéraud F, Viau M, et al. Meat processing and colon carcinogenesis: cooked, nitrite-treated, and oxidized high-heme cured meat promotes mucin-depleted foci in rats. Cancer Prev Res. 2010;3:852–64. 10.1158/1940-6207.CAPR-09-0160. 10.1158/1940-6207.CAPR-09-0160 PMC2931773 20530708

[41] Steed J, Sanchez F, Mcguinness M, Stahl-zeng J. A rapid method for quantifying nitrosamine compounds with qualitative confirmation using the SCIEX QTRAP® 6500+ LC-MS/MS System. Framingham, MA, USA; 2018. https://sciex.com/content/dam/SCIEX/pdf/tech-notes/all/A-Rapid-Method-for-Quantifying-Nitrosamine-Compounds-with-Qualitative-Confirmation.pdf. Accessed 22 July 2025.

[42] International Council for Harmonisation of Technical Requirements for Pharmaceuticals for Human Use (ICH). ICH harmonised guideline: bioanalytical method validation and study sample analysis M10. Geneva, Switzerland; 2022. https://www.ich.org/page/multidisciplinary-guidelines. Accessed 28 July 2025.

[43] U.S. Food and Drug Administration (FDA). ORA Lab Manual Vol. II - Methods, method verification and validation (ORA- LAB.5.4.5). Food Drug Adm Off Regul Aff Qual. 2023. https://www.fda.gov/media/73920/download. Accessed 24 Oct 2025.

[44] Deenadayalan L, Pillai S, Baghla R, Jones E, Nandita E. Low-level quantification of 10 mutagenic nitrosamine impurities in acyclovir. Featuring sensitive quantification of nitrosamine impurities using the QTRAP 6500 + system. Framingham, MA, USA; 2018. https://sciex.com/tech-notes/pharma/qa-qc/low-level-quantification-of-10-mutagenic-nitrosamine-impurities-0. Accessed 22 July 2025.

[45] Deda O, Gika HG, Wilson ID, Theodoridis GA. An overview of fecal sample preparation for global metabolic profiling. J Pharm Biomed Anal. 2015;113:137–50. 10.1016/j.jpba.2015.02.006. 25812436 10.1016/j.jpba.2015.02.006

[46] Wojnowski W, Tobiszewski M, Pena-Pereira F, Psillakis E. AGREEprep – analytical greenness metric for sample preparation. TrAC-Trends Anal Chem. 2022;149:116553. 10.1016/j.trac.2022.116553.

[47] González-Martín R, Gutiérrez-Serpa A, Pino V, Sajid M. A tool to assess analytical sample preparation procedures: sample preparation metric of sustainability. J Chromatogr A. 2023;1707: 464291. 10.1016/j.chroma.2023.464291. 37582319 10.1016/j.chroma.2023.464291

[48] Nowak PM, Wojnowski W, Manousi N, Samanidou V, Plotka-Wasylka J. Red analytical performance index (RAPI) and software: the missing tool for assessing methods in terms of analytical performance. Green Chem. 2025;27:5546–53. 10.1039/d4gc05298f.

[49] Fuente-Ballesteros A, Martínez-Martínez V, Ares AM, Valverde S, Samanidou V, Bernal J. Violet innovation grade index (VIGI): a new survey-based metric for evaluating innovation in analytical methods. Anal Chem. 2025;97:6946–55. 10.1021/acs.analchem.5c00212. 40139928 10.1021/acs.analchem.5c00212 PMC12131222

