# Supplementary material for: The development of an analytical method to evaluate the nitrosamine profile in cooked ham with different preservatives and in rat feces fed with them
Source: Anal Bioanal Chem. 2025 Nov 23;418(1):219–35. doi: 10.1007/s00216-025-06214-2 (PMC12774933; doi:10.1007/s00216-025-06214-2)
Supplement: Supplementary file 1 — Supplementary Material 1 Fig. S1 Evaluation of the acidic medium used during the ultrasound-assisted extraction step on the sensitivity of NAs. Fig. S2 Evaluation of the fecal sample amount on the peak intensity of quantitative ion of each NA using ultrasound-assisted extraction. Fig. S3 Chromatograms obtained for a cooked ham sample spiked with 100 ng g−1 NAs. Table S1. Calibration curves for the determination of NAs in cooked ham and feces samples. Table S2. P-value of the PERMANOVA test in the cooked ham matrix. Table S3. P-value of the PERMANOVA test in the fecal matrix. Table S4. P-value of Fisher exact test to evaluate the association between the presence of NAs in cooked ham and in feces. Table S5. P-value of Z-test to compare the detection rate of nitrosamines specific to a matrix (cooked ham or feces) (PDF 478 KB) [file 216_2025_6214_MOESM1_ESM.pdf]

The development of an analytical method to evaluate the nitrosamine profile in cooked ham with different preservatives and in rat feces fed with them

Claudia Giménez-Campillo [1] · Yolanda Guerrero-Núñez [1] · Natalia Campillo [1] · Natalia Arroyo-Manzanares [1] · Isidro Guillén [2,3] · Pascuali Vizcaíno [2,3] · Carlos de Torre-Minguela [2,3] · Pilar Viñas [1,*]

[1] *Department of Analytical Chemistry, Faculty of Chemistry, Regional Campus of International Excellence "Campus Mare Nostrum", University of Murcia, E-30100 Murcia, Spain*

[2] *Cátedra PROSUR de Biotecnología de Alimentos, University of Murcia, E-30100 Murcia, Spain*

[3] *Department of Research and Development. PROSUR S.L., Av. Francisco Salzillo, P/27-2, San Ginés, E-30169 Murcia, Spain*

* Corresponding authors at: Department of Analytical Chemistry, Faculty of Chemistry, Regional Campus of International Excellence "Campus Mare Nostrum", University of Murcia, E-30100 Murcia, Spain (P. Viñas).

E-mail addresses: pilarvi@um.es (P. Viñas).

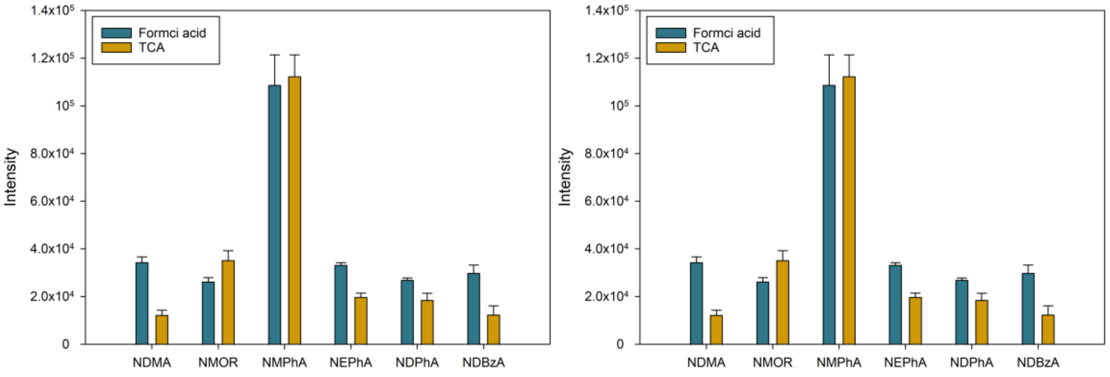

Fig. S1 Evaluation of the acidic medium used during the ultrasound-assisted extraction step on the sensitivity of NAs.

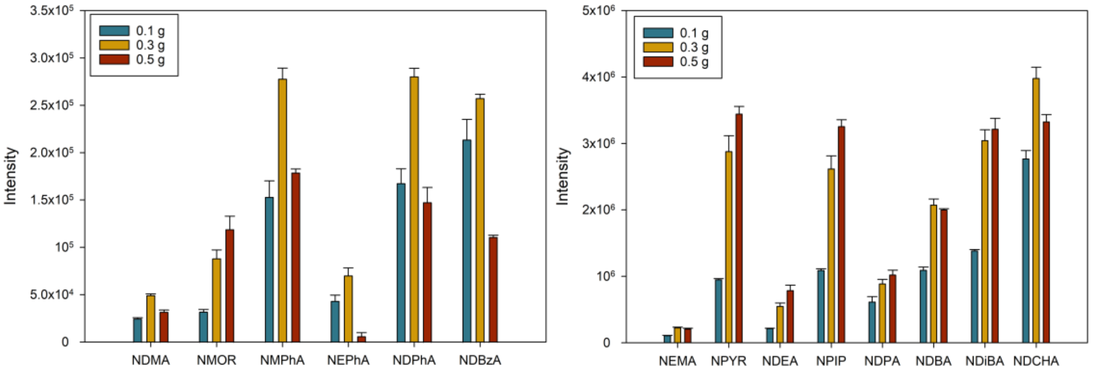

Fig. S2 Evaluation of the fecal sample amount on the peak intensity of quantitative ion of each NA using ultrasound-assisted extraction.

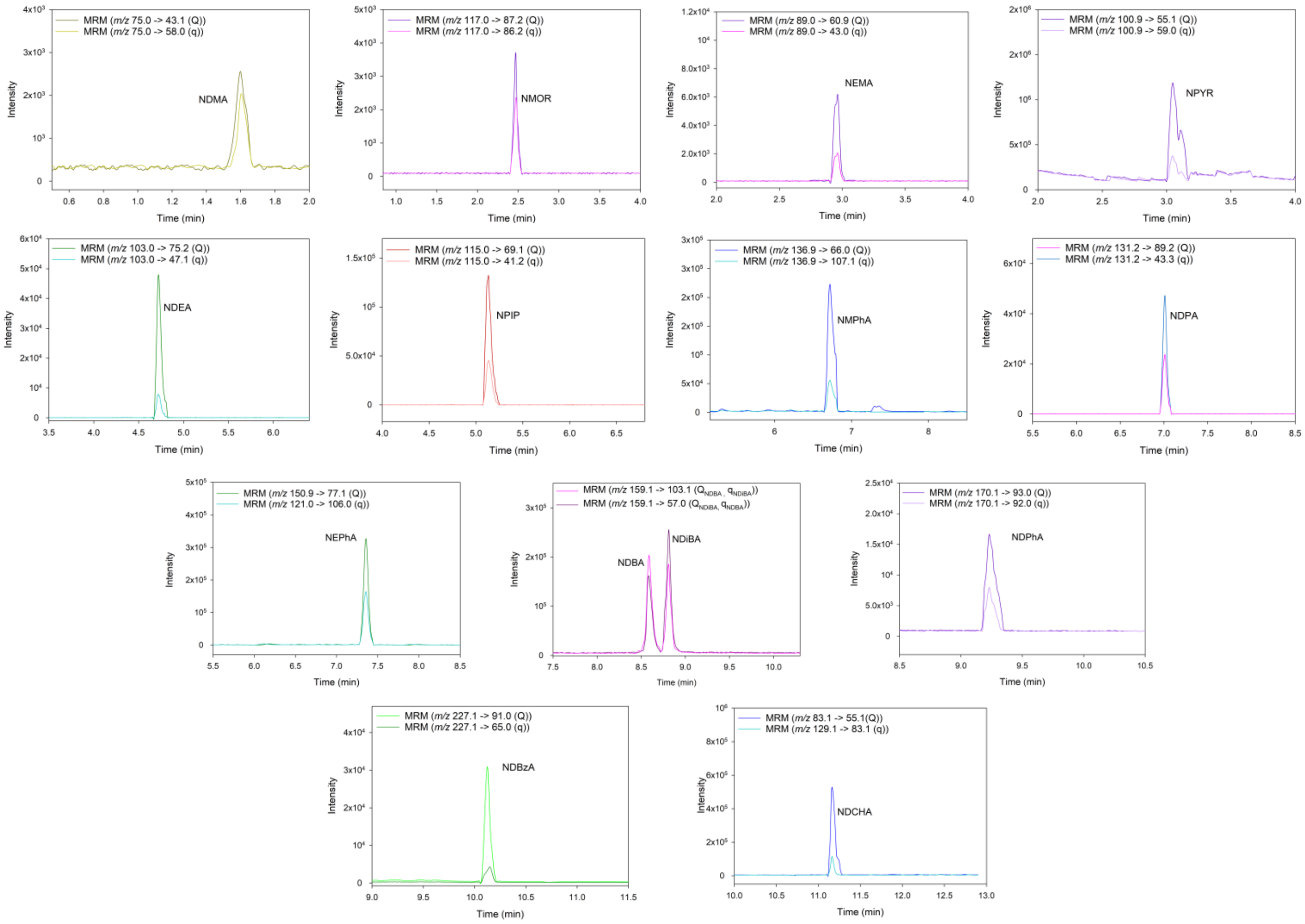

Fig. S3 Chromatograms obtained for a cooked ham sample spiked with 100 ng g^{-1} NAO.

Table S1. Calibration curves for the determination of NAs in cooked ham and feces samples

	Cooked ham samples		Feces samples	
Analyte	Slope, g ng^{-1}	R^2	Slope, g ng^{-1}	R^2
NDMA	116	0.9957	186	0.9915
NMOR	180	0.9977	294	0.9968
NEMA	797	0.9982	1536	0.9931
NPYR	5876	0.9992	9520	0.9938
NDEA	1820	0.9978	2345	0.9977
NPIP	5662	0.9994	9583	0.9961
NMPhA	2543	0.9948	6429	0.9939
NDPA	2776	0.9960	3821	0.9963
NEPhA	344	0.9939	383	0.9921
NDBA	5545	0.9932	11258	0.9978
NDiBA	4655	0.9931	10613	0.9990
NDPhA	487	0.9946	1087	0.9961
NDBzA	533	0.9917	814	0.9926
NDCHA	2645	0.9958	7023	0.9926

Table S2. *P-value* of the PERMANOVA test in the cooked ham matrix

	Nitrite-free ham	Nitrite ham	Ham with polyphenols
Nitrite-free ham		0.4591	0.1316
Nitrite ham	0.4591		0.4808
Ham with polyphenols	0.1316	0.4808	

Table S3. *P-value* of the PERMANOVA test in the fecal matrix

	Nitrite-free diet	Nitrite diet	Diet with polyphenols
Nitrite-free diet		0.16	0.0022
Nitrite diet	0.16		0.0027
Diet with polyphenols	0.0022	0.0027	

Table S4. *P-value* of Fisher exact test to evaluate the association between the presence of NAs in cooked ham and in feces

	Nitrite-free	Nitrite	Polyphenol	All together
NMPhA	0.467	0.010	0.091	0.002
NDBA	0.400	-	0.541	0.116
NDiBA	0.167	0.205	0.081	< 0.001

Table S5. *P-value* of Z-test to compare the detection rate of nitrosamines specific to a matrix (cooked ham or feces)

	Nitrite-free	Nitrite	Polyphenol	All together
NDMA	0.060	0.139	0.064	0.005
NDEA	-	0.040	-	0.039
NDPA	0.060	-	-	0.077
NPYR	0.136	0.352	-	0.077
NEPhA	0.136	0.139	0.064	0.005
NDPhA	0.136	0.005	-	0.003
